# Enhancing integrated palliative care: what models are appropriate? A cross-case analysis

**DOI:** 10.1186/s12904-017-0250-8

**Published:** 2017-11-28

**Authors:** Sheila Payne, Rachael Eastham, Sean Hughes, Sandra Varey, Jeroen Hasselaar, Nancy Preston

**Affiliations:** 1 International Observatory on End of Life Care, Division of Health Research, Lancaster University, Lancaster, LA1 4YG UK; 2 Division of Health Research, Lancaster University, Lancaster, LA1 4YG UK; 3 Department of Anaesthesiology, Pain and Palliative Medicine, Radboud University Medical Center, Nijmegen, The Netherlands

**Keywords:** Integrated care, Palliative care, Qualitative research, End-of-life care, Physician-patient relations, Continuity of care, Hospice, Advanced disease, Delivery of health care, Primary health care

## Abstract

**Background:**

Effective integration between hospices, palliative care services and other local health care services to support patients with palliative care needs is an important international priority. A previous model suggests that integration involves a cumulative stepped process of engagement with other organisations labelled as ‘support, supplant or supplement’, but the extent to which this model currently applies in the United Kingdom is unknown. We aimed to investigate accounts of hospice integration with local health care providers, using the framework provided by the model, to determine how service users and healthcare professionals perceived palliative care services and the extent of integration experienced.

**Methods:**

Longitudinal organisational case study methods were employed using qualitative serial interviews (interval 3 months) with patients and family carers focusing on how services responded to their needs; and group interviews with health professionals. Data were audio-recorded, transcribed verbatim, and analysed by qualitative content analysis and combined across data sources.

**Results:**

The study focused on four hospices in northern England, including 34 patients (diagnosis: 17 cancer, 10 COPD, 7 heart failure), 65% female, mean age 66 (range 44–89), 13 family carers of these patients (48% partners), and 23 health care professionals. While some care fell short of expectations, all patients reported high levels of satisfaction and valued continuity of care and efficient information sharing. All hospices supported and supplemented local providers, with three hospices also supplanting local provision by providing in-patient facilities.

**Conclusion:**

UK hospices predominantly operate in ways that support and supplement other providers. In addition, some also supplant local services, taking over direct responsibility and funding in-patient care. They all contributed to integration with local services, with greater blurring of boundaries than defined by the original model. Integrated care offers the necessary flexibility to respond to changes in patient needs, however, constraints from funding drivers and a lack of clear responsibilities in the UK can result in shortfalls in optimal service delivery. Integrating hospice care with local healthcare services can help to address demographic changes, predominantly more frail older people, and disease factors, including the needs of those with non-malignant conditions. This model, tested in the UK, could serve as an example for other countries.

## What is already known

- The importance of effective integration of hospices with other local health and social care organisations is increasingly recognised.
- There is considerable diversity in the nature of integration between hospices and other local health and social care organisations.

## What this paper adds

- This study examined the extent to which hospice integration could be regarded as a cumulative stepped process involving the support, supplement and supplanting of the services of other local organisations.
- Evidence that hospices predominantly support and supplement local providers, with some supplanting them when they take over direct responsibility and funding for in-patient care.
- Collaborative integrated initiatives across organisational interfaces demonstrated some blurring of boundaries, calling for further conceptual thinking about the implementation of integrated hospice care.

## Implications for practice, theory or policy

- Across all health and social care sectors, integration offers opportunities to improve patient transitions between care settings, transcend traditional boundaries of service provision, and enhance continuity of care.
- Systems to improve information transfer between service providers, and with patients and families, are crucial.
- Cross boundary care provision challenges current clinical and organisational concepts requiring further research to test models of integrated palliative care.

## Background

Palliative and hospice care is internationally recommended as an essential part of healthcare systems [1]. Globally, there is diversity in hospice care provision. For example, hospice programmes in the USA that are reimbursed by Medicare are typically delivered on a home care basis, often very near the end of life [2]. In contrast, in Germany, hospices typically offer patients longer term nursing care in residential settings, which are distinct from specialist palliative care units located in hospitals [3]. In the United Kingdom (UK), most hospices emerged in the latter decades of the twentieth Century as independent organisations funded largely by local charitable donations. Their foundation and growth was outside central healthcare planning or the British National Health Service (NHS). The 220 British hospices, which are providers of specialist palliative care, are generally located in purpose-built accommodation, with in-patient beds, day care, home care and associated services [4]. International comparative studies indicate that palliative care in the UK is rated highly [5, 6]. However, the UK along with many other countries is facing demands from an ageing population, with multiple morbidities which have numerous agencies involved in delivering care for increasing numbers of patients. This is within the context of economic pressures from reduced national resources available for the NHS and social services, and some limitations to local philanthropy.

**Fig. 1:**
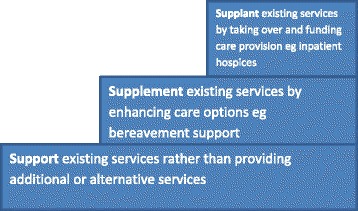
Stepped model of care provision

Sometime ago, based upon research conducted in New Zealand and the UK, Payne proposed a cumulative stepped model of integration between hospices and local health and social care providers [7]. Figure 1 indicates the three cumulative processes of engagement: 1) Support of existing services by providing resources/equipment or specialist consultations to assist others to provide care rather than providing alternative care (for example, loaning specialist equipment such as Age Concern); 2) Supplement existing local services by enhancing care options such as bereavement support and contributing specialist palliative care (for example, complementary therapy services); and 3) Supplant existing services by taking on medical responsibility and funding care provision such as in-patient hospices (for example, independent hospices).

In recent years, there have been calls for greater integration of palliative care across all care settings to facilitate continuity of care, improve quality of life and reduce inappropriate hospital admissions for patients [8]. The Integrated Palliative Care in cancer and chronic conditions (InSup-C) study that started in 2012, aimed to investigate promising practices in Europe and to formulate requirements for effective palliative care integration across the continent [9]. Within the InSup-C project, the following working definition for integrated palliative care was empirically developed: ‘Integrated palliative care involves bringing together administrative, organizational, clinical and service aspects in order to realize continuity of care between all actors involved in the care network of patients receiving palliative care. It aims to achieve quality of life and a well-supported dying process for the patient and the family in collaboration with all the caregivers, paid and unpaid’, but remains untested [10].

We aimed to investigate accounts of hospice integration with local health care providers, using the framework provided by the model in Fig. 1, to determine how service users and healthcare professionals perceived palliative care services and the extent of integration experienced.

In addition, we seek to investigate practices associated with care as experienced by patients, family carers and health professionals which promote or limit integration.

## Methods

### Design of the study

We employed a qualitative interpretative approach to a secondary analysis of data drawn from the InSuP-C study. We selected the case study methodology described by Yin as ‘multiple embedded’ case studies because of its suitability for real-world situations where the researchers have little control over confounding or other variables [11]. ‘Multiple’ refers to the selection of more than one case site for the purpose of data collection – each hospice constituting a ‘case’. Our analysis was ‘embedded’ within a number of sources of data (patients, family carers, health professionals) from within and across the case study sites. This method is regarded as appropriate for palliative care contexts [12, 13]. A full account of the InSuP-C methodology is published [9].

### Setting

Hospices in Northern England were identified based on the inclusion criteria shown in Table 1 [9].

**Table 1:** Inclusion criteria for organisational cases

• the hospice is part of an established local palliative care collaboration; • the collaboration must contain at least two different organizations; • a hospital can be part of that collaboration; • collaborating healthcare professionals must provide direct patient care (not only an advisory function); • the collaboration has a multidisciplinary background (professionals of different professions must be involved, e.g. physician (specialist, GP), nurse (specialist), social worker, Allied Health Professional, spiritual worker, complementary therapist, others); • the collaboration aims to provide palliative care for one or more patient diagnostic groups (advanced COPD/heart failure/cancer).	

### Participants

Purposive sampling was used to identify patients living at home in the three diagnostic groups with advanced disease. Patients were initially approached by professionals within the hospices directly involved in patient care. Family carers were identified by patients. Patients and carers, if available, were interviewed twice by the researcher (SH) following written, informed consent. Multidisciplinary health professionals associated with each ‘hospice case’ were invited to participate together in group interviews. Participation was self-selected and all who wished to participate did so.

### Data collection

In-depth, face-to-face interviews with patients were conducted at two time points, 3 months apart, to capture change over time, and were conducted between June 2014 and July 2015. Interviews were either dyadic (patient and researcher) or combined with patient and family carers. They were mostly conducted in patients’ homes and typically lasted 60–90 min. While following a topic guide, semi-structured interviews were also flexible to enable participants to describe their experiences of care provision (Appendix 1 – interview guides). Prompt cards were used to assist participants’ recall of salient aspects and to identify priorities. The follow up interview considered how services had responded to patient and carer needs during the intervening 3 months and in particular to any changes or increase in need for service provision.

Group interviews with health professionals (employed by the hospice and local providers) were facilitated by SH and NP and were conducted at each hospice. A guided conversation was designed to elicit staff experiences of general integrated palliative care and typically took between 90 and 120 min. In addition, field-notes were collected by the research team during site visits to build up a contextual picture of each hospice ‘case’. Data were collected from July 2014 until October 2015.

### Data analysis

All interviews and group discussions were audio-recorded and transcribed verbatim. The participants were allocated a numerical code to ensure anonymity. Analysis was conducted in two phases: 1) initial thematic analysis used a coding structure that had been developed for the wider international study [14]. SH coded transcripts and NP reviewed a random sample of them. Contested codes or themes were discussed and resolved. 2) Subsequently, secondary analysis of professional group interviews, patient and carer interviews was carried out by SV and RE specifically for this paper usingYin’s ‘cross case synthesis’ [11]. This method was selected as our research contained multiple organizational cases (4 hospices) which meant that the findings were likely to be more robust than only having a single case [11]. We also undertook a comparison of the case study data in relation to the cumulative stepped model [7]. This combined an examination of data across the four cases and comparison with the model in Fig. 1. Direct quotations have been selected to highlight typical responses, and are indicative of the diversity of experiences reported. To establish rigor, we adhered to COREQ guidelines [15].

## Results

We start by describing the individual participants and the four hospice cases, before comparing and contrasting the cases drawing upon the model [7]. Diagnostic characteristics of the patients (*n* = 34), carers (*n* = 13) and health professional (*n* = 23) are presented in Table 2. Half of the patients had cancer, and over half were female. They had a mean age of 66 years (range 44–89 years). All identified themselves as White British, except one British Asian; 12 patients lived alone. Of the overall sample, five patients died before a second interview could take place, two moved into residential care and one patient was too ill to participate. Thirteen family carers consented to participate and completed a baseline interview. Of these, two withdrew from the study due to their partners’ increasing care needs. Four family carers completed a bereavement interview in the weeks following the death of the patient. Nearly half of carers were partners of patients. In the follow-up interviews, participants reported services responded positively to advancing disease with some exceptions. One person with COPD had a key staff member leave and he reported that services deteriorated. One person became rapidly ill and was admitted to acute hospital care, then spent a weekend in pain because no health professional would provide adequate analgesia. Two people moved into residential care, one to ease family burden and one because his home care service became inadequate for his increased need.

**Table 2:** Patient, family carer and health professional sample

Case study site	Patients	Family carers	Health professionals
Hospice A	Cancer 5	7	6
Urban location	COPD 4		
	Heart failure 4		
Hospice B	Cancer 7	3	6
Rural location	COPD 1		
	Heart failure 0		
Hospice C	Cancer 1	2	5
Urban location	COPD 2		
	Heart failure 2		
Hospice D	Cancer 5	1	6
Urban location	COPD 2		
	Heart failure 1		
Total	34	13	23

Twenty three health professionals (21 female) took part in four group interviews, one at each hospice. The majority were qualified nurses from community, day hospice and in-patient settings. Five were physicians including three GPs and two palliative medicine consultants. There was one occupational therapist, physiotherapist, social worker and chaplain. The mean length of time in their current post was 6.5 years (range 1–19), and mean period since qualification 21 years (range 5–42). Table 3 provides the characteristics of the four selected hospices. We describe the services delivered by each ‘organisational case’ (hospice) and the perspectives of patients and families, including an indicative account from a patient with cancer, heart failure and COPD to illustrate perceptions of integrated palliative care.

**Table 3:** Description of the four hospice cases

Site	Est.	In-patient services	Income^a^	Other services	Referral	Geography	Funding	Other
Hospice A	1985	20 beds	£8.8 million	Day therapy Community palliative care team Hospice at home Lymphodema clinic Complementary therapy service Spiritual care for patients and families Counselling services for patients/families and bereaved 24 h advice line for professionals and patients/carers The hospice part-funds a social worker (important because of the area’s socio-economic factors)	By professionals for any condition deemed to be palliativeor for patients who have end of life care needs	Runs services across 2 areas: an urban area and a rural area. The urban area has a population of 140,000 and is an area of significant economic deprivation with, for example, high levels of ill-health, smoking, obesity and alcohol consumption.	70% charitable 30% NHS	Service Level Agreements^b^ with the two local Clinical Commissioning Groups (CCGs) in their area. Soon to change to a more formalised contractual arrangement. No formal agreements with other health and social care agencies.
Hospice B	1987	None - Remote/detached services that consolidates care from: 5 local hospitals; Nursing and residential care homes; Local GPs; District nurses; Community matrons; Macmillan nurses (Clinical Nurse Specialists in Palliative Care)	£1.1 million	Home nursing (mainly delivered by health care assistants) A lymphedema service Family and bereavement support (mostly delivered by trained volunteers under the supervision of a paid coordinator). One-to-one and group support Complementary therapies (mostly delivered by trained volunteers supported by a small number of paid staff).	Professional and self-referral for all end of life conditions.	Rural location. The area is remote from large towns, sparsely populated and has high levels of social and economic deprivation. Hospice B represents a home care service.	25% NHS 75% charitable	Service Level Agreement with the CCG. Renegotiated each year. Collaboration with a range of other agencies, such as health and social care agencies in the local area, is not formally agreed and occurs on an ad hoc basis as required.
Hospice C	1985	17 beds	£9.8 million	Home care service Day therapy Bereavement support Complementary therapy support Volunteer led patient and family support	By professionals for any condition deemed to be palliative	Situated in a mixed urban and rural area. 2011 census cites the population of the town and environs as ~138,000. Deprivation is lower than the average for England (2015, Public Health England) although rates for alcohol and smoking related harm are higher than the English average. The area is well connected with several major road and rail links, locally.	30% NHS 70% charitable	Service Level Agreement with the CCG in their area. No formal agreements with other health and social care agencies with whom the agency collaborates in the local area. One consultant works between the hospice and hospital.
Hospice D	2001	16 beds	National charity income	Day therapy Drop-in sessions Complementary therapies Art group Intravenous infusions and blood transfusions Religious and spiritual support Outpatient appointments Heart Failure Programme COPD (and other pulmonary diseases) specific service	MND patients are referred automatically at diagnosis. All other patients become involved at the point at which it is deemed they have a specialist or complex need that cannot be met by general services, as defined by the referring professional. Self-referrers are asked to get their GP (or other involved professional) to refer on their behalf.	Serves a major conurbation (Population estimated as 530,000 in 2016). This city is close to other large cities in an inland area of the North of England. The city has a large, longstanding south Asian population. It is reportedly in the top 20 most deprived districts in England and the trend is worsening. Life expectancy is lower than the average for England for both men and women and significant health inequalities persist (2015, Public Health England).	30% NHS 70% from a local fundraising and main national charity.	Part of a clinical network which includes hospital, community, hospice and academic representation from specialist and generalists palliative care. There are no formal agreements with other health and social care agencies in the local area.

^a^2016 data acquired from the charities commission website

^b^These do not constitute formal contracts. The CCGs are the groups that distribute NHS funds by commissioning healthcare services in a local geographical area

### Hospice A – Service description

Hospice A provided comprehensive in-patient and home care. The hospice supplemented NHS funded primary care. Hospice A delivered services across an urban location and surrounding rural area. A smooth integration of care was assured by, for example, weekly multidisciplinary team meetings chaired by the specialist palliative care physician. All relevant local organisations were co-ordinated and followed standardised procedures using paper based medical records that were similar throughout the local care system. Hospice A partially funded a social worker to respond to the needs of the local community which suffered significant economic deprivation. The role of key professionals was important within the patient’s care network. For example, Community Matrons (senior community based nurses) cared for patients with complex, chronic conditions such as advanced COPD. They knew their patients well, had up-to-date information and could transfer this information to other professionals. Such key contacts were important to enhance patient care as increasing numbers of professionals became involved in the support network:

‘I think what’s confusing for patients and relatives […] is there are that many people involved in the care, when there is a problem who do I ring?’ (Hospice Staff Nurse, Hospice A, Professional Group Interview).

### Hospice A - patient/carer perspectives

Most patients and carers were generally positive about the care at Hospice A, particularly when they had a named professional. However, patients sometimes reported fragmented service delivery and poor patient-professional communication as illustrated below:

‘You try and get an appointment with your (GP), it’s four weeks away. When you’re in excruciating pain on a bed and you’re screaming out, four weeks is… it might as well be four months away’…..

‘People [are] too scared to cross each other’s territories […] there’s no one central person to go to. If we had one person to go to regardless of what medical problem you had and they then made the decision where you’d to go, it would be far easier than being passed from pillar to post’ (Hospice A,P08, Cancer).

#### Male COPD patient

Small team supporting patient with two key professionals working in close collaboration, Evidence of regular communication between key workers. Long established relationship with specialist palliative care nurse, in particular. Criticism of pulmonary follow up services as not worth much except for access to oxygen supply and maintaining nebuliser. (AP01).

#### Male cancer patient

General perception that care is not well connected and some things are missing: e.g. Macmillan (specialist nurse) support; home care plan not being implemented by home care workers who apparently do not stay for the agreed length of time; too many different home care workers prevented relationship building and negatively impacted on continuity of care. He valued the hospice day care as a social space and for combatting aloneness (as he lived alone). (AP07).

#### Male CHF patient and female carer

Community matron was the key organising professional. Patient perceived good inter- professional connection and communication. This patient’s family carer also reported that the Community Matron was a key professional and ‘lynch pin’ of care provision. She described a clear demarcation between home and hospice care. (AP05 + ACO2).

### Hospice B – Service description

Hospice B provided specialist palliative care services at home, without in-patient facilities, which supplemented NHS primary care. Hospice B served a dispersed population living in a rural location. Staff were proud that they were ‘probably more integrated than a lot of teams’:

‘I think we need to work harder at working together [...] *because* we cover such a huge geographical area’ (Hospice B Professional group interview).

However, integration could fail if communication and planning were insufficient:

‘The care agency didn’t know that this man was home and by the Monday tea-time he had died [...] He had two days of really … no care’ (Hospice B Professional group interview)

### Hospice B – Patient/carer perspectives

Hospice B was typically described by patients and carers as peripheral to their concerns about GPs and access to hospital services such as oncology. Hospice B was therefore perceived as supportive by providing extra services like complementary therapy and group support sessions.

#### Male COPD patient and female carer

Patient was very happy with all the services he received and could not identify any unmet needs. However, this patient’s family carer identified lack of response from their GP as problematic. This was resolved by the assignment of a ‘designated’ GP. The family carer described the benefits of having a regular staff member who was someone who knew where they are coming to at night as they lived in a remote, rural location. She valued honest communication gently delivered by a key professional as too many different professionals can lead to contradictory advice and confusion for patient and carer. (BPO2 + BCO1).

#### Female cancer patient

Patient reported poor GP continuity of care; some apparent uncertainty about which professional is responsible for what; with the GP taking a ‘back seat’ when the specialist nurse is leading: complicated by a lack of GP continuity. (BP03).

### Hospice C – Service description

Hospice C provided in-patient beds, home care, day care, out-patient clinics and bereavement support, and therefore appeared to ‘supplant’ local services. Hospice C was a hub for specialist palliative care and also delivered a home care service which supplemented many aspects of general palliative care services provided by primary care in the locality.

### Hospice C – Patient/carer perspective

As with Hospice A, lack of continuity of care was problematic for some patients and carers at Hospice C. The following example showed how integration appeared to fail at times.

‘It’s no good one person knowing more than the other, or that person not being aware of this particular aspect, or that person being out of the loop. If you all know the same things then you’ve a better quality of treatment and more chance of success’ (Hospice C, P01, COPD).

#### Female COPD patient

Reported good communication between GP and Community Matron; but no evident communication between a care agency and anyone else in the care network (CP02).

#### Female cancer patient

“I *think it’s important to have an efficiently-run service and the more these people can work together, the more efficient the service becomes.”* She reported starting to see more integrated working between GP surgery and District Nurses. (CP05)

#### Female CHF patient and male carer

They were very appreciative of the professionals involved in their care network but did not see any cardiology specialists regularly for follow-up: ‘*it’s all done through the GP’*. The patient had been an inpatient at Hospice C for a week and had recently been attending Day Therapy once a week for 12 weeks. In the previous year, she attended Day Therapy once a week for 16 weeks, but it has now been reduced for all patients. For this patient, Hospice C was regarded as a very important support and she trusts her GP who has a key role in her care network. (CP03 + CCO1).

### Hospice D – service description

Hospice D provided inpatient beds, home care, day care, out-patient clinics and bereavement support. Hospice D supplemented NHS medical services. This hospice and other organisations in this large urban area used the same locality-specific electronic medical records system facilitating communication and the sharing of patient information. Professional participants reported that it contributed to the coherence of service delivery. Hospice D had, over the past decade, worked with specialist community heart failure nurses and community matrons (with a primary responsibility for those with chronic conditions in the community, particularly COPD) to develop targeted services for these patients and their family caregivers. These included information and activity sessions for heart failure patients and their carers delivered in the hospice day care unit, and the provision of a ‘Breath Better’ course for people with COPD. This programme aimed to reduce breathlessness and improve illness mastery and quality of life using education, exercise, medication management and individual consultations.

The heart failure service was a collaborative initiative with the local health authority that employed specialist heart failure nurses.

### Hospice D – patient/carer perspective

Hospice D was praised for its coherence by some patients and carers who benefitted from an electronic information system:

‘Efficiency of electronic information system (highlighted); experiencing siloed care and a lack of holism from medical professional’ (Hospice D P01, cancer).

#### Male COPD patient

He reported proactive contact from his GP and enjoyed the social interactions during Day Therapy at hospice D. Day to day care mainly managed by a daughter who lived nearby and regular visits from the district nursing service. No specialist COPD nursing input received so the hospice day therapy clinical input was regarded as key for this person. This led to concerns from the patient and reportedly, from family members, about the restricted length of day therapy service provision (limited to 12 weeks) and what would happen thereafter. (D P03).

#### Female cancer patient

She liked to come to hospice D because it functions as a one stop shop. However, she describes having to do a lot to coordinate her own care by contacting a range of different services and health professionals. She sometimes felt too unwell to do this. She complained about the lack of proactive care from Macmillan (specialist palliative care) nurses and feels she has to direct her own care. (DP04).

#### Female CHF patient and female carer

An 8-week mindfulness course (accessed through Hospice D) has helped her to get through some unpleasant heart tests. In the first interview, this patient mentioned that she had ‘picked’ one GP who she would ask to see for continuity of care. But this GP has now left and she doesn’t know the other GPs. She reported that Hospice D has been fantastic. Unlike her GP surgery, they answer her calls, staff are responsive and she feels they are there for her if she needs anything. This patient’s family carer reported on lack of communication and sharing medical information between cardiologist and GP. (DP08 + DCO1).

#### Comparative analysis

Hospices predominantly operate in ways that support and supplement other providers. In addition, three of the four hospices also supplanted the care provided by local services when the patient was admitted for an inpatient stay. As patients and carers were interviewed at home, their primary experience of hospice provision was as a supplement to care provided by their GP and primary care. Overall they were very positive about the care they received regardless of the nature of integration.

Unlike the original study in New Zealand [7], we found no evidence of hospices *only* offering ‘support’ to other organisations. Hospice B, unlike the other hospices, did not provide in-patient beds and largely supplemented NHS primary care [7]. When this hospice was first established, it worked with local community hospitals and nursing homes to provide inpatient care if necessary, while maintaining patients at home where possible. All hospices were similar in that they accepted referrals of patients with any palliative needs (not just cancer). All hospices were largely funded by local charitable donations and to a less extent NHS contracts resulting in some uncertainty about income and challenges to forward planning. Hospice services were delivered in association with the local NHS health authorities based on service level agreements, rather than formal contracts. These were regularly (re)negotiated and are an example of the financial insecurity experienced by hospices [16].

In Hospices A, C and D, day care was valued by patient and carers but its continuity, which was time limited, was problematic for some. The use of day care appeared to be especially valued by those with non-cancer. Referral back for further hospice care was responsive to patients’ fluctuating needs.

## Discussion

The main aim of this study was to investigate accounts of hospice integration with local health care providers, using the framework provided by the model in Fig. 1, to determine how service users and healthcare professionals perceived palliative care services and the extent of integration experienced.

We found that patients and carers across *all* hospices valued continuity of care, integrated working and a named professional as a point of contact. Staff highlighted the benefits of joint patient information systems and multidisciplinary meetings to facilitate integration. While there have been international policy calls for greater integration [1], there has been a lack of clarity about implementation, and evidence of inequity for those in rural areas and with non-cancer [17]. Since undertaking the study, new recommendations from the WHO have been published outlining a model of comprehensive, person-centered, and integrated palliative care [18]. While the model advocates networking, cooperation, and shared care capacity, it provides no evidence that it has been tested empirically.

A cumulative stepped model of hospice integration with local services showed that all hospices supported and supplemented other local services, and some even supplant other services, by providing in-patient facilities. We identified that the boundaries of the original model have become blurred. The extent of integration in the study hospices was more flexible than suggested and appears to be mediated by factors that include: patients’ diagnoses and disease trajectories; availability of family carers; ethos of the hospice; availability of staff and diversity of services (which is largely contingent on funding); and geographical location of the patients.

Over the years, there has been a number of shifts in thinking about the nature of palliative care, about who it should be delivered to and when. This partly reflects an ageing population, increasing consumer expectations and greater pressures on the NHS. Older patients normally present with multi-morbidity which is associated with more hospital deaths [19, 20]. Hence hospice services need to work in conjunction with local agencies to meet the needs of patients with multi-morbidities, as demonstrated by a clinical trial [21].

A key area of integration was information sharing about patients to allow continuity and tailored care which are central to ensuring positive experiences [22, 23]. Sharing of clinical information in the NHS has been a long standing concern [24] however, Hospice D, for example, participated in a local electronic shared medical records initiative. Systems that relied on paper records, meetings and telephones, enabled relationship building with other teams but pressure from increasing numbers of patients makes this challenging to administer.

The role of GPs in the UK is under threat, with a national shortage, resulting in concerns about inadequate care [25]. GPs are responsible for care co-ordination but in practice may seldom see patients [25]. In accordance with other British and European research, specialist nurses and hospice teams appeared to work closely together but this may leave GPs feeling deskilled [8, 25]. However some GPs maintained their coordination role and facilitated continuity of care, as exemplified in Germany, The Netherlands and Belgium [8].

### Strengths and limitations

Using self-report data from patients, carers and health professionals enabled us to elicit their experiences. However, the use of purposive, rather than random, sampling, raises the possibility for selection bias. Our focus on four hospices does not provide a comprehensive overview, as regional differences may distort patterns of integration. Moreover, the original model was proposed almost 20 years ago based largely on data collected in New Zealand where hospices, at that time, predominantly provided cancer care. Further conceptual modelling of what integration means in palliative care contexts is warranted.

## Conclusions

A complex picture of hospice integration in the UK emerged highlighting the needs of patients with non-cancer diagnoses and multi-morbidities. This means that greater integration by hospices in the UK is required to work with, rather than replace, local providers, with more clarity in managing cross organisational information sharing and allocation of co-ordination roles and responsibilities. Priorities for integrated working include a single information system and a skilled named professional to coordinate care and form meaningful relationships. It is vital that future hospice and palliative care services in the UK are designed in ways that integrate care across local providers and take heed of factors that patients identify as undermining best care.
